# Implementation of a CRISPR-Based System for Gene Regulation in *Candida albicans*

**DOI:** 10.1128/mSphere.00001-19

**Published:** 2019-02-13

**Authors:** Elvira Román, Ioana Coman, Daniel Prieto, Rebeca Alonso-Monge, Jesús Pla

**Affiliations:** a Departamento de Microbiología y Parasitología, Facultad de Farmacia, Instituto Ramón y Cajal de Investigaciones Sanitarias (IRYCIS), Universidad Complutense de Madrid, Madrid, Spain; Carnegie Mellon University

**Keywords:** CRISPR, *Candida albicans*, Gal4, RNA scaffold, catalase, gene activation, gene repression, genetic tool

## Abstract

Clustered regularly interspaced short palindromic repeat (CRISPR) methodology is not only an efficient tool in gene editing but also an attractive platform to facilitate DNA, RNA, and protein interactions. We describe here the implementation of a CRISPR-based system to regulate expression in the clinically important yeast Candida albicans. By fusing an allele of Streptococcus pyogenes Cas9 devoid of nuclease activity to a transcriptional repressor (Nrg1) or activator (Gal4), we were able to show specific repression or activation of the tester gene *CAT1*, encoding the cytosolic catalase. We generated strains where a 1.6-kbp upstream regulatory region of *CAT1* controls the expression of the green fluorescent protein (GFP) and demonstrated the functionality of the constructs by quantitative PCR (qPCR), flow cytometry, and analysis of sensitivity/resistance to hydrogen peroxide. Activation and repression were strongly dependent on the position of the complex in this regulatory region. We also improved transcriptional activation using an RNA scaffolding strategy to allow interaction of inactive variants of Cas9 (dCas9) with the RNA binding protein MCP (monocyte chemoattractant protein) fused to the VP64 activator. The strategy shown here may facilitate the analysis of complex regulatory traits in this fungal pathogen.

**IMPORTANCE** CRISPR technology is a new and efficient way to edit genomes, but it is also an appealing way to regulate gene expression. We have implemented CRISPR as a gene expression platform in Candida albicans using fusions between a Cas9 inactive enzyme and specific repressors or activators and demonstrated its functionality. This will allow future manipulation of complex virulence pathways in this important fungal pathogen.

## Abstract (precis)

CRISPR technology is a new and efficient way to edit genomes, but it is also an appealing way to regulate gene expression. We have implemented CRISPR as a gene expression platform in Candida albicans using fusions between a Cas9 inactive enzyme and specific repressors or activators and demonstrated its functionality. This will allow future manipulation of complex virulence pathways in this important fungal pathogen.

## INTRODUCTION

CRISPR (clustered regularly interspaced short palindromic repeats) are short sequences present in microbial genomes that allow immune recognition of foreign DNA (1, 2). In recent years, the mechanism by which this occurs has enabled numerous applications, especially in genome editing of higher eukaryotes, given its simplicity compared to other methods involving zinc finger proteins and transcription activator-like effector nuclease (TALEN) enzymes (3–5). Although CRISPR systems are diverse and widely distributed among bacteria and archaea (6, 7), those most frequently used in genetic manipulation rely on Cas9, a Streptococcus pyogenes-derived type II enzyme that recognizes sequences complementary to a small RNA. This so-called guide RNA (gRNA) requires not only Watson-Crick pairing but also an appropriate stretch of nucleotides (termed the protospacer adjacent motif [PAM]) adjacent to the target sequence. The PAM for S. pyogenes Cas9 is NGG, allowing several guides to be found in almost any DNA sequence. Following recognition, Cas9 cleaves DNA via its RuvC and HNH nuclease domains, and, in the presence of an appropriate template, it can be used by the cell to repair the cleaved allele by homologous recombination. CRISPR has been implemented in a wide range of taxons (8) and has proven a useful tool in fungal research, not only for pathogenic fungi (9, 10) but also for yeasts, for which several tools are already available (11, 12).

Candida albicans is a clinically relevant diploid pathogenic fungus that is commonly found as a harmless commensal of humans but which is able to cause severe diseases among immunocompromised individuals. The development of genetic tools in this fungus is important for the discovery of novel virulence genes and antifungal agents. A CRISPR editing system was recently implemented in C. albicans through the construction of a C. albicans codon usage-adapted version of the S. pyogenes Cas9 endonuclease (13). Those authors created knockout strains simultaneously altered in both chromosomal alleles, thus circumventing the use of two different markers or a marker recycling strategy (14–16). Given the high efficiency of the nuclease, even double-disruption events in two different genes were simultaneously accomplished. Stable integration in the genome is not necessary, as introduction of PCR products devoid of replicons provides transient expression that is functional for gene deletions (17), as shown also in other pathogenic *Candida* species (18). The system has been improved through increased gRNA production via an alternative promoter/posttranscriptional processing scheme (19). Gene drives (20) have been also implemented in C. albicans and, combined with the availability of haploid C. albicans strains (21), have allowed the easy construction of deletion sets of mutants via mating (22).

While CRISPR has proven extremely useful in strain construction, it has also gained interest as a platform to facilitate interactions among RNA, DNA, and proteins. Inactive variants of Cas9 (dCas9) have been generated by mutagenesis in their nuclease domains (D10A and H840A) but retained their ability to target a specific sequence. Protein fusions between dCas9 and cargo protein enable activation or repression of transcription in eukaryotes (23, 24), visualization of specific chromosomal locations (25), or even editing of genomes without cleaving DNA (26). Activation or repression of a given gene is particularly useful in the dissection of biological process in pathogens, especially as the nature of CRISPR allows multiplexing (27–29). We describe here the implementation of CRISPR-based modulation of transcription in C. albicans. By fusing a catalytically dead version of Cas9 to the transcriptional activation domain of Gal4 and/or VP64 and the Nrg1 repressor, we demonstrated specific gene regulation of the catalase *CAT1* gene, a result with important applications for genetic dissection of biological processes in this fungus.

## RESULTS

### Generation of a catalytically inactive Cas9 protein in Candida albicans.

**FIG 1:**
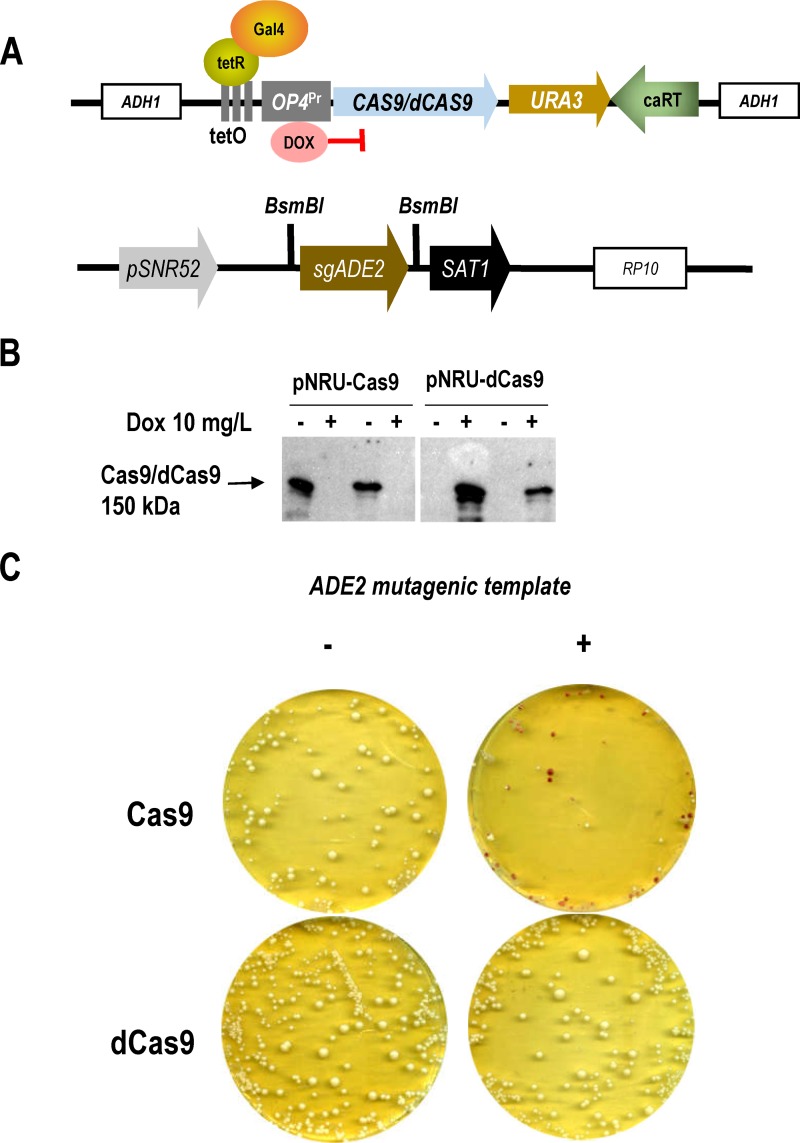
Construction and validation of an inactive Cas9 allele. (A) CRISPR constructs used to validate dCas9 in C. albicans. The first one (top) integrates *CAS9* or *dCAS9* at the *ADH1* genomic region, and expression of Cas9 alleles is under the control of the TET^OFF^ system (repressed by doxycycline [DOX]). The second one (pV1090, bottom) (13) integrates at *RP10* and is used to clone the desired guide (*ADE2* in this case) by digestion with BsmBI under the control of the *SNR52* promoter. (B) Protein extracts from strains expressing either Cas9 (pNRU-Cas9) or dCas9 (pNRU-dCas9) after 24 h of growth in YPD medium at 37°C in the absence (-) or presence (+) of doxycycline (Dox) (10 mg/liter) were analyzed by Western blotting. A band of 150 kDa corresponding to Cas9 or dCas9 was detected with antiCas9 antibody. (C) *ADE2* mutagenesis by CRISPR is achieved only when Cas9, *ADE2* sgRNA, and a repair mutagenic template (+) are cotransformed in C. albicans as determined by the appearance of red colonies.

In order to use CRISPR for gene regulation instead of genome editing, we generated an RNA-guided DNA-binding protein by eliminating the ability of Cas9 to cleavage DNA. We introduced the D10A and H840 point mutations that abolish the RuvC-like and HNH nuclease activity by overlapping PCR (see Materials and Methods), generating a C. albicans-adapted version of dCas9. We cloned both *CAS9* and *dCAS9* in plasmids under the control of the TET^OFF^ system and integrated them in C. albicans at the *ADH1* genomic region in wild-type strains (Fig. 1A). As expected, in the presence of doxycycline (Dox) in the medium, the expression of *CAS9* or *dCAS9* was completely blocked and no protein was observed in whole-cell extracts (Fig. 1B). To demonstrate the absence of nuclease activity of dCas9, we compared the abilities of both Cas9 and dCas9 to induce mutagenesis of *ADE2*. We integrated at the *RP10* locus a single guide RNA (sgRNA) against *ADE2* to abolish the activity of *ADE2*, thus rendering red cells and colonies (13). As shown in Fig. 1C, when we simultaneously expressed *CAS9* and the guide under inducing conditions (without Dox), red colonies were generated when a complementary repair template with a stop codon was added. However, no red colonies appeared in cells expressing dCas9, indicating that no DNA cleavage and therefore no repair had occurred via homologous directed repair (Fig. 1C). We therefore used this allele for subsequent studies.

### Implementation of CRISPR as a tool for transcriptional activation in C. albicans.

**FIG 2:**
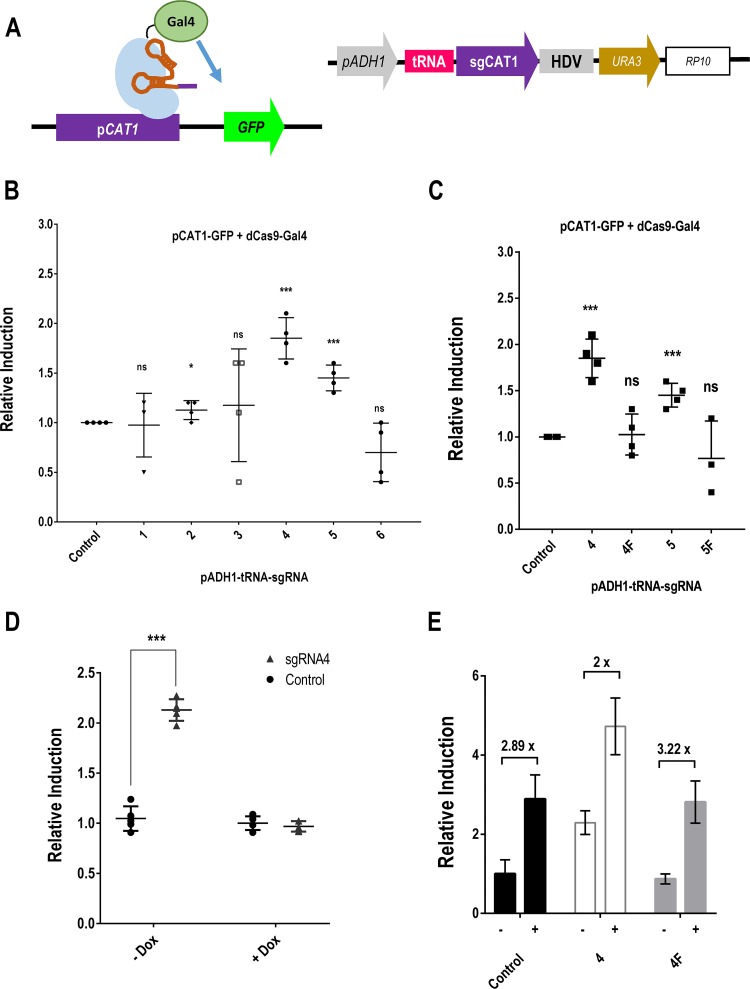
dCas9-Gal4-mediated activation of transcription in C. albicans. (A) Diagram of the dCas9-Gal4 fusion as an RNA guided DNA binding protein to the *CAT1* promoter. sgRNAs were expressed using the pADH1-tRNA scheme (19). (B) Relative MFI induction ratios of GFP detected by flow cytometry in cells coexpressing dCas9-Gal4 and the indicated guides after 24 h of growth at 37°C in SD medium. The data are displayed as means ± standard deviations of results from 3 independent experiments performed with different transformants; “Control” refers to a strain with vector but no guide. (C) Fold induction of the MFI from different sgRNAs compared to the corresponding strain without guide (Control). (D) GFP Relative MFI induction ratio of the indicated strains after 24 of growth at 37°C in the presence (+) or absence (-) of doxycycline (Dox). The data are displayed as means ± standard deviations of results from 3 independent experiments and were normalized to the control strain without guide in the absence of Dox. (E) Stationary-phase cells were treated or not with 5 mM hydrogen peroxide (H_2_O_2_) for 2 h. Relative MFI induction ratios are represented in cells coexpressing dCas9-Gal4 and sgRNA4 (4) or sgRNA4F (4F). Data are normalized to the values for the vector without any guide (Control) in the absence of hydrogen peroxide. *, *P* < 0.05; ***, *P* < 0.001; ns, not significant.

In order to test whether dCas9 can be used as a modular RNA-guided platform to specifically recruit CRISPR transcriptional activators (CRISPRa) to DNA, we chose the *CAT1* gene, which encodes the cytosolic catalase in C. albicans. A 1.6-kbp region upstream of the methionine starting codon was fused to the green fluorescent protein (GFP) gene and integrated at the *ARD1* locus (see Materials and Methods) to generate the tester strain pCAT1-GFP, where *CAT1* expression can be easily checked by both microscopy and flow cytometry (FC) (see Fig. S1A and B in the supplemental material). An *in silico* analysis of this DNA using the Chop-Chop server (http://chopchop.cbu.uib.no) identified sequences susceptible to be used as CRISPR guides (20 nucleotides [nt] followed by a PAM). Different targets were chosen, and guides were cloned under the control of the pADH1-tRNA promoter (19), as it has been suggested that expression of the sgRNA flanked by a 5′ tRNA and transcribed by a strong RNA polymerase II *ADH1* promoter increases sgRNA production, which could be a bottleneck in CRISPR/Cas9 editing (19). We constructed vectors expressing dCas9 fused to transcriptional activator Gal4 (Fig. 2A) (30), as this activator has been successfully used for engineering a C. albicans adapted reverse *trans*-activator (rtTA) in doxycycline-dependent induced regulation (31). The dCas9-Gal4 chimera was introduced into the pCAT1-GFP strain using the pADH1-tRNA guide production scheme (19).

10.1128/mSphere.00001-19.1 FIG S1 (A) Genetic arrangement of the tester strain pCAT1-GFP (top). Phase contrast (top) or fluorescence (bottom) photographs of the parental RM1000 strain and pCAT1-GFP after 24 h of growth in minimal SD medium and the corresponding flow cytometry histogram are shown. (B) Cells pf the pCAT1-GFP strain were diluted in SD minimal medium, and the MFI was followed for 24 h. Representative flow cytometry profiles (left) and time course of the MFI (right) are shown. C) Schematic representation of the targets chosen for *CAT1* promoter profiling. Numbering refers to the ATG, A therefore representing 1. Download FIG S1, TIF file, 1.2 MB. Copyright © 2019 Román et al. 2019 Román et al. This content is distributed under the terms of the Creative Commons Attribution 4.0 International license.

**FIG 3:**
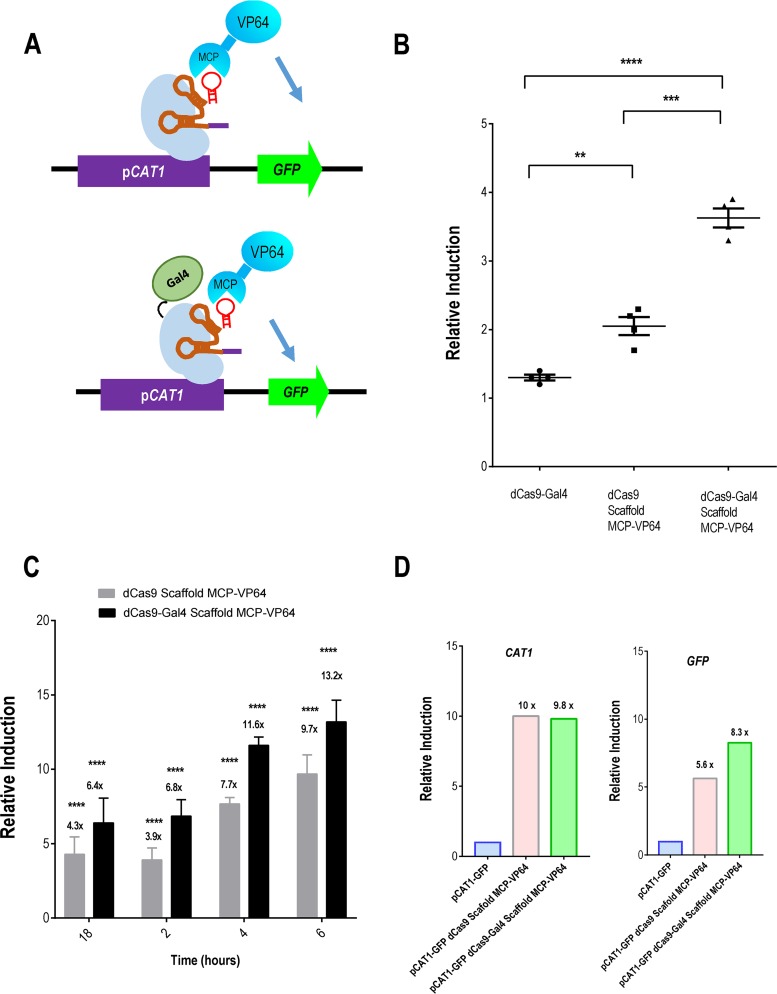
RNA-mediated scaffolding increased transcriptional activation. (A) Design of the modular RNA structure encoding the MS2 domain used to recruit RNA binding protein MCP fused to transcriptional activator VP64 at the *CAT1* promoter and scheme of the dual-activator system that uses Gal4 fused to dCas9 and scRNA-MCP-VP64 modules guided to the *CAT1* promoter. (B) Fold increase (induction relative to the tester strain, pCAT1-GFP) of the GFP MFI detected by flow cytometry of the indicated strains after 24 h of growth in SD medium at 37°C. Values represent means ± standard deviations of results from least 4 independent experiments. **, *P* < 0.01; ****, *P* < 0.0001. (C) Overnight (18-h) cultures of the indicated strains were diluted in prewarmed fresh SD medium and incubated at 37°C. At different time points (2, 4, and 6 h), samples were processed and analyzed by flow cytometry; the fold increase of GFP MFI (induction relative to the tester strain, pCAT1-GFP) is represented. (D) Overnight cultures of the indicated strains were diluted in prewarmed fresh SD medium, incubated at 37°C, recovered at 5 h, and processed for qPCR. The fold increase of the mRNA levels of *CAT1* and *GFP* (relative induction) obtained from the indicated strain compared to control strain pCAT1-GFP is represented.

An analysis of the relative GFP levels of induction (determined by comparing the mean fluorescence intensity [MFI] of the population to that of cells without a guide) of strains after 24 h of growth in SD medium (2% glucose, 0.5% ammonium sulfate, 0.17% yeast nitrogen base and amino acids) at 37°C revealed that guides 4 and 5 gave the clearest results (Fig. 2B), while the rest of the guides showed few (guide 2) or no statistically significant differences. As shown in Fig. 2C, the MFI ratio increased only when the activator (dCas9-Gal4) was coexpressed with guide 4 compared to strains without any sgRNA (control). Guide 4 hybridizes at positions −197 to −183 with respect to the starting methionine. We included in these experiments complementary target guides (4F and 5F) devoid of a PAM site as internal controls; those controls, as expected, showed no increase (MFI values were 74 ± 15 for the vector, 88.6 ± 12.3 for control guide 4F, and 144.6 ± 9.7 for guide 4, thus showing ∼2-fold induction) (Fig. 2C). Guide 5 (sgRNA5F) also led to GFP expression but to a lesser extent (∼1.5×), confirming the relevance of the positioning of the complex along the promoter to modulate transcription. In other to confirm that regulation was dependent on the activator module, we compared the GFP MFI results for cells growing in the presence or absence of doxycycline (Fig. 3D). As expected, the addition of the drug resulted in MFI levels similar to those seen with the control strain lacking the activator module, while in its absence, the level was found to have increased ∼2-fold to 2.5-fold. We also analyzed whether the CRISPRa system could interfere with the canonical *CAT1* promoter regulation. When stationary-phase cells from strains expressing the activator module and different sgRNAs were subjected to hydrogen peroxide for 2 h and GFP expression was quantified (Fig. 3E), we observed similar increases of MFI (∼2-fold-to-3-fold-higher) in response to the oxidant in all strains. Therefore, despite induction of activation by dCas9, the promoter was still sensitive to its canonical triggering stress.

### A dual-CRISPR system enhances transcriptional gene activation.

In order to improve gene activation, we implemented the RNA scaffolding system recently described in mammalian cells and Saccharomyces cerevisiae (32). This system is based on incorporating a single RNA hairpin into the 3´ end of the sgRNA; this additional module is recognized by its corresponding RNA binding protein, which can then be fused to transcriptional activators or repressors, allowing the recruitment of additional transcriptional regulators to a specific DNA region. To generate these scaffold RNA constructs, we fused the viral RNA sequence of the phage MS2 3´ end to the sgRNA in the original gRNA production scheme (13) (called “scRNA” [small cytoplasmic RNA]). We also developed a chimera between the MS2 RNA-binding protein (monocyte chemoattractant protein [MCP]) and the VP64 transcriptional activator (see Materials and Methods), both chemically synthesized and codon adapted for C. albicans (33) (see Materials and Methods) (Fig. 3A). The system was tested in the same strain (pCAT1-GFP) with dCas9 under the control of the TET^OFF^ promoter and the scRNA with the corresponding activator module. After 24 of growth in SD medium at 37°C, the analysis of GFP MFI by flow cytometry showed an approximately 1.6-fold increase in the MFI of cells expressing scRNA using guide 4 compared to the strain without a guide or with the 4F control guide (not shown) (2.05 ± 0.26 compared to 1.3 ± 0.08, *P* = 0.0016) (Fig. 3B). We reasoned that recruiting different activators would increase transcriptional activation and therefore combined the two strategies, i.e., a direct fusion of Gal4 to dCas9 and the RNA scaffold to recruit the MCP-VP64 module. Comparing the levels of activation of the reporter gene, we observed that the dual system showed increases of approximately 3.5-fold relative to the control strain (MFI of 3.6 ± 0.28, *P* < 0.0001) and 1.7-fold relative to strains expressing only the scRNA (*P* = 0.0002) (Fig. 3B).

Interestingly, we observed that induction levels were higher when MFIs were determined during the exponential phase of growth. In fact, an analysis of the *CAT1* promoter revealed that the MFI of the pCAT1-GFP tester strain varied along the phase of growth, with the 24-h cells showing high catalase levels compared to the exponentially growing cells (Fig. S1B). We therefore analyzed the phase dependence patterns of our constructs. Stationary cultures from the tester strain, dCas9 with scRNA-MCP-VP64, and dCas9-Gal4 with scRNA-MCP-VP64 (all expressing guide 4) were diluted in prewarmed fresh SD medium, and GFP was determined by flow cytometry at different time points. As shown in Fig. 3C, MFI values were increasing in both scaffold constructs during the log phase, reaching a maximum at 6 h postdilution and always being higher with the dual-activation system (Fig. 3C). In order to validate the levels detected by flow cytometry, we quantified mRNA by quantitative PCR (qPCR) analysis of both the *CAT1* and *GFP* genes (Fig. 3D). Comparing the activation induced by scRNA in either the dCas9 or the dCas9-Gal4 strains 5 h after dilution from stationary-phase growth, we observed that the level of *CAT1* was ∼10-fold higher than that of the pCAT1-GFP strain in both strains, while GFP expression was higher when the dual system was used (8.3-fold versus 5.6-fold). Therefore, the dual-activation system is able to activate gene expression in C. albicans.

**FIG 4:**
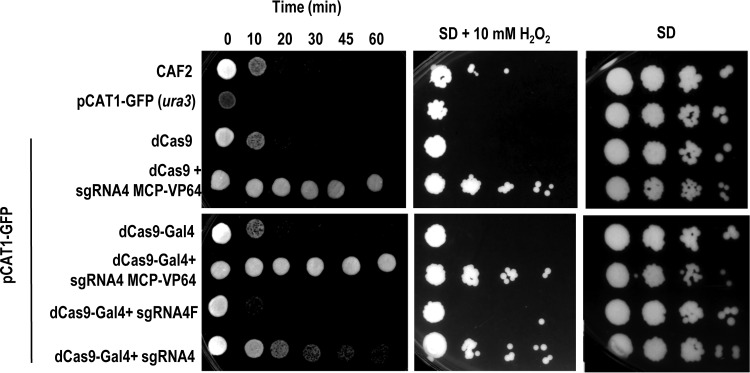
Susceptibility to hydrogen peroxide. Cells of the indicated strains from cultures grown 4 h at 37°C after dilution from stationary phase were serially diluted 10-fold and plated on SD media containing hydrogen peroxide (H_2_O_2_) at the indicated concentration or subjected to 100 mM H_2_O_2_ in liquid SD medium and spotted at different times (indicated in minutes) onto YPD plates. Images were taken after 24 h of growth at 37°C.

We tested whether dCas9-mediated transcriptional activation would also (as expected) increase transcription of native *CAT1* by testing the susceptibility of the indicated strains to hydrogen peroxide. We did two types of analyses: a standard drop assay on oxidant-supplemented plates and an assay of loss of cell viability in liquid medium upon challenge with a lethal concentration of oxidant. For these experiments, we chose cells growing in logarithmic phase at 4 to 5 h after dilution in fresh SD medium from overnight cultures. As shown, the cells that coexpressed the dCas9-Gal4 activator with guide 4 were more resistant to hydrogen peroxide than the wild-type CAF2 strain cells and the controls expressing the flipped guide (guide 4F) (Fig. 4). These results corroborate the data from the transcriptional analysis. As expected, those strains that directed a dCas9-Gal4-MCP-VP64 chimera via guide 4 scRNA to the promoter were more resistant to hydrogen peroxide than those expressing only one of activator module dCas9-Gal4 and activator module dCas9-MCP-VP64 (Fig. 4). Therefore, CRISPR-mediated gene activation in C. albicans has functional consequences that can be detected by phenotypic analysis.

### Implementation of CRISPR as a tool for gene repression in C. albicans.

**FIG 5:**
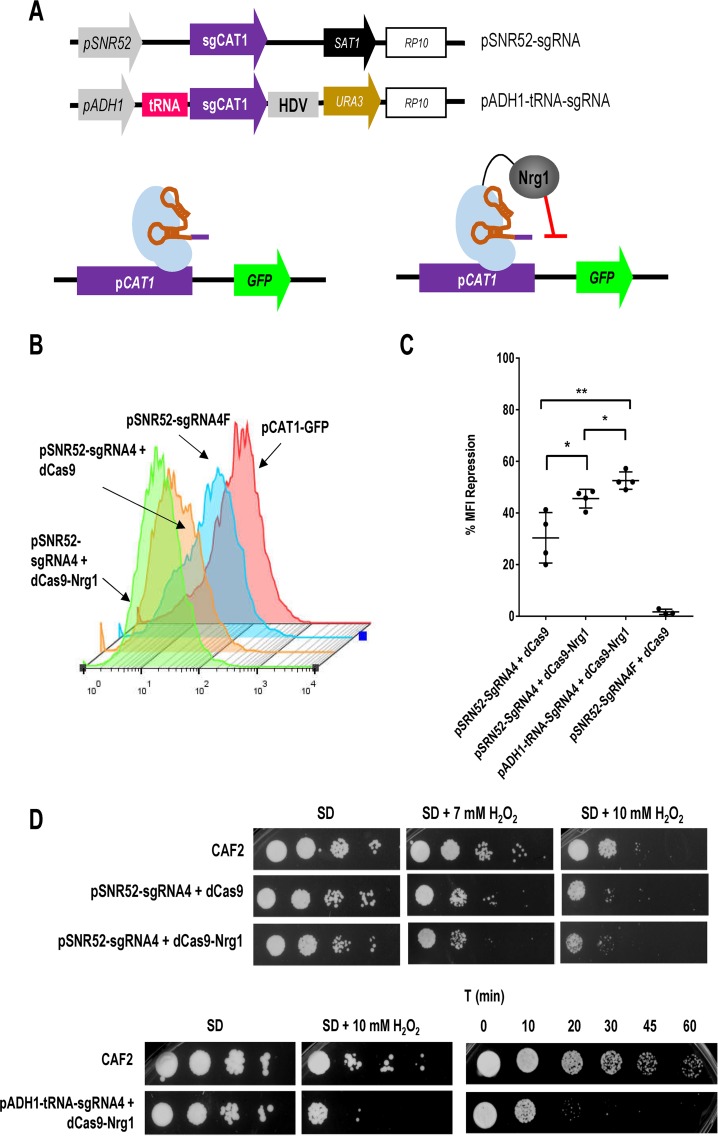
dCas9-mediated repression of transcription. (A) Genetic constructs used to test repression. Design of the CRISPR interference systems requires the expression of dCas9 or of a dCas9 fused to the repressor Nrg1 and a sgRNA that targets the nuclease death protein to the *CAT1* promoter in a tester strain which harbors the promoter fused to GFP. (B) Flow cytometry profiles of the indicated strains after 24 h of growth in SD medium at 37°C. (C) Percentage of transcriptional repression of the indicated strains normalized to the MFI level of the corresponding control strain which expresses only the sgRNA4 or sgRNA4F in the pCAT1-GFP background. Values represent means ± standard deviations of the results from at least three measurements from three independent transformants. (D) Oxidant susceptibility of cells expressing *dCAS9* or *dCAS9-NRG1* and sgRNA4 in both guide systems. Cells of the indicated strains from the stationary phase were serially diluted 10-fold and plated on SD media containing hydrogen peroxide (H_2_O_2_) at the indicated concentration or subjected to 75 mM H_2_O_2_ in liquid SD medium and spotted at different times (indicated in minutes) onto YPD plates. Images were taken after 24 h of growth at 37°C.

To check if dCas9 could be used to repress transcription, we used similar constructs with different markers (*URA3* or *SAT1*) to express *dCAS9* under the control of the TET^OFF^ system (Fig. 5A) and generated the corresponding strains harboring guide 4, as this guide showed the most evident influence on regulation.

The analysis of the GFP signal by flow cytometry under conditions of full expression (minimal SD medium without doxycycline), using the pSNR52 promoter and guide 4, showed a 30.4% ± 9.8% blockage. We also tested the effect of incorporating full-length Nrg1, a cognate repressor in C. albicans (Fig. 5B and C), which was added to the C terminus of dCas9 (see Materials and Methods). In this case, the level of blockage with dCas9-Nrg1 was 45.5% ± 3.6%. As expected, no blockage (1.7% ± 1%) was obtained when the control flipped version (guide 4F) was used (Fig. 5C). The results obtained by using the pADH1-tRNA scheme (19) were slightly better (53% ± 3.4%) than those obtained by using pSNR52 (13). These results demonstrate the ability of the CRISPR interference (CRISPRi) system to repress gene expression in Candida albicans.

We tested whether blockage of *CAT1* expression resulted in increased oxidant susceptibility using either standard drop assays on SD plates with hydrogen peroxide or kinetics of loss of viability in liquid media supplemented with the oxidant. The corresponding strains generated with both systems were grown for 24 h in SD medium at 37°C and subjected to oxidative stress. When dCas9 was guided by guide 4, the cells became more susceptible to hydrogen peroxide than the cells expressing only dCas9 or the cells of the wild-type host strain CAF2 (Fig. 5D, top panel). In this experiment, no differences were observed using the Nrg1 repressor, as dCas9-Nrg1 behaved similarly to dCas9. The phenotypes seen with the two strategies were similar, and expression of the guide using pADH1-tRNA resulted in either growth defects in plates or a decay of cell viability kinetics (Fig. 5D, bottom panel). No defects in growth were observed when control guide 4F was used (data not shown). These results demonstrated that CRISPR can be specifically used to reduce gene expression in C. albicans.

## DISCUSSION

The peculiar role of S. pyogenes Cas9 as an RNA-and-DNA-interacting protein enables biological approaches where it can be used as a scaffold to facilitate these biological interactions. We demonstrated here that the CRISPR system may be used to modulate gene transcription in C. albicans by using catalase *CAT1* as a tester gene. We chose this gene since, on the basis of our observations (34, 35), moderate changes in its amount result in clear phenotypes regarding hydrogen peroxide sensitivity.

We demonstrated here that CRISPR can be used both to activate and to repress transcription in C. albicans. Negative regulation of transcription (CRISPR interference [CRISPRi]) has been demonstrated previously in bacteria by targeting the gRNA-dCas9 complex at different places within a monomeric red fluorescent protein (mRFP) test gene (23). Those authors were able to show approximately 300 repression as well as strand-specific positional effects at both the transcription initiation and elongation levels (23). Such values are far from those attained in S. cerevisiae, which showed much modest levels. For example, a decrease of 18× was observed previously for a *TEF1-GFP* construct (24), and that level can be increased to 50× with the Mxi1 transcriptional mammalian repressor, which has been reported to interact with the yeast Sin3 acetylase. More-modest (2× to 3×) reductions or even an absence of such reductions has been reported previously in yeast (36), indicating that the simple positioning of the gRNA-dCas9 complex does not efficiently interfere with transcriptional initiation/elongation. In an attempt to circumvent such an outcome, yeast and mammalian repressors have been fused to dCas9. For example, in Yarrowia lipolytica, the use of dCas9-Mxi1 was previously shown to result in 10× repression (37) whereas the use of dCas9-KRAB was previously shown to result in 2.5× repression and the use of a tripartite Ume6-Mig1-Tup11 domain to result in 5× repression in comparison to Mxi1 (38). In our case, we used Nrg1 since this protein is a well-known repressor of hyphal genes (39, 40). The fold repression observed was in the range of 40% to 60% (Fig. 5C), which is similar to what is observed in other yeasts. We avoided the use of filamentation-inducing conditions in the analysis of catalase expression, as we used full-length Nrg1 and the dCas9-Nrg1 construct could interfere with the expression of hypha-specific genes. However, this would be easy to engineer in the future using the Nrg1 repressor domain and/or alternative repressor modules (see accompanying paper by L. Wensing, J. Sharma, D. Uthayakumar, Y. Proteau, et al.).We used different transcriptional activation domains and strategies to increase expression. We used the Gal4 activation domain (indicated as Gal4 in the figures) to create dCas9-Gal4 chimeras, obtaining a 2-fold-to-3-fold doxycycline-specific increase in GFP transcription. These values were obtained when sgRNAs were expressed under the control of the pADH1-tRNA promoter and were slightly higher than those obtained when guides were expressed under the control of the pSNR52 promoter with the system described previously by Vyas et al. (13), which is in concordance with what has been described previously (19). Although these values are still modest, they are close to those determined in a similar study in S. cerevisiae using a pCYC1-GFP construct (36). As Gal4 shows strong effects and is routinely used in C. albicans to achieve a high level of expression (31), we think that the level seen here may represent a limitation of the experimental setup. We thus combined this strategy, in a dual system (Fig. 3A), with a RNA scaffolding strategy (32) in which a scaffold RNA is used to recruit transcriptional activators via a RNA interacting protein. Using four tandem copies of the herpes simplex virus protein 16 (VP64 activator), we were able to increase this value to approximately 4× (Fig. 3B). Such increases were moderate when cells were analyzed in stationary phase but reached approximately 10× to 13× during the log phase of growth (Fig. 3C), demonstrating that activation was increased when different activator modules were recruited (Gal4 plus VP64). Studies in mammalian cells have shown drastic increments of the signal; however, these values are usually obtained only when several copies of the target sequence are placed in tandem upstream the tester gene. Increasing the number of available gRNA 5′ to the gene significantly increases the gene reporter output (24, 32, 36). Despite these current limitations, we showed the critical role of the specific region of the dCas9-AD complex in modulation, as has been also observed in other recent studies (36). We studied up to 6 different guides along with the *CAT1* promoter and demonstrated that guide 4, which lies at positions −197 to −183 approximately 100 bp upstream of the most probable TATA box, was the most efficient for CRISPRa (Fig. 2B), suggesting that this spacing is the spacing best suited for transcriptional activation, at least for *CAT1*. We also present evidence of effects at the endogenous native *CAT1* locus, as shown in Fig. 4 and 5, where the effect on endogenous *CAT1* expression correlated with a pattern of resistance/susceptibility to hydrogen peroxide, thus providing evidence for the interaction of the Cas9 complex at the native *CAT1* gene.

Future improvements of the system will surely involve studies on regulatory regions of other genes to determine the precise spacing required for optimal effects on transcription as well as for developing new dCas9 chimeras. As dCas9 production does not seem to be limiting (19), coexpressing several guides will simplify the analysis of complex pathways such as those shown in S. cerevisiae (32). This technology will also enable genome-wide analysis of regulatory regions in C. albicans, which will surely facilitate understanding the regulation of virulence in this important pathogen.

## MATERIALS AND METHODS

### Strains and growth conditions.

The strains used are listed in Table S1 in the supplemental material. Cells were grown at 37°C in YPD medium (1% yeast extract, 2% peptone, 2% dextrose) or complete SD medium (2% glucose, 0.5% ammonium sulfate, 0.17% yeast nitrogen base and amino acids) unless otherwise stated. The analysis of susceptibility/resistance to different compounds was performed using the standard drop test as follows. Cultures grown at 37°C from stationary-phase cells were adjusted to 2 × 10^7^ cells/ml, serially 10-fold diluted, and deposited (5 µl) onto solid SD plates supplemented (or not) with hydrogen peroxide at different concentrations. Plates were incubated at 37°C for 24 h and were then scanned. When necessary, doxycycline was added to either liquid or solid medium at 5 to 10 or 20 mg/liter, respectively. For flow cytometry (FC) analysis, cells were recovered 24 h after growth at 37°C in SD complete medium (stationary phase) or at the indicated times after dilution in fresh SD medium (log phase) from stationary-phase cultures. Cells were fixed in 1% phosphate-buffered saline (PBS)-formaldehyde and washed twice with PBS previous to fluorescence-activated cell sorter (FACS) analysis performed using a Guava cytometer (Millipore).

10.1128/mSphere.00001-19.2 TABLE S1 List of strains used in the study. We include the name referred in the manuscript, the lineage or the laboratory where the strain was originated, and the genotype. Download Table S1, XLSX file, 0.01 MB. Copyright © 2019 Román et al. 2019 Román et al. This content is distributed under the terms of the Creative Commons Attribution 4.0 International license.

### Molecular biology procedures and construction of plasmids.

All plasmids and oligonucleotides are listed in Table S2 and Table S3, respectively. C. albicans *CAS9* (*CaCAS9*) was amplified with primers Up-Cas9 and Rev-Cas9 (Table S3) using pV1093 (kindly provided by Vyas et al.) (13) as the template. *CadCAS9* was obtained by overlapping PCR to introduce the corresponding mutations using primer pair dCas9-o1 and dCas9-o2 and primer pair dCas9-o3 and dCas9-o4. Both the *CaCAS9* and *CadCAS9* amplicons were introduced in the intermediate pGEM-T vector (Promega), digested with SalI*/*NotI, and then inserted in the pNRU expression vector, with *URA3* used as a selection marker (41), to generate pNRU-CAS9 and pNRU-dCas9 plasmids. Both plasmids were finally digested with KpnI*/*SacII to force homologous recombination at the *ADH1* locus. pNIM1RX-dCas9 vectors, with *SAT1* used as a selection marker, were generated by the insertion of the SalI/NotI dCas9 fragment into the construct that had been digested previously with the same pair of enzymes, and pNIM1RX-RFP vector was generated by replacing the 5´ *ADH1* XbaI/SacI fragment from pNIM1R-RFP (42) with a 1.630-bp XbaI/SacI fragment from the pNIMX vector (43), containing the 5´ *ADH1* and the *TDH3* promoter. Homologous recombination at the *ADH1* locus was forced after enzymatic digestion with KpnI*/*SacI. Transformants were selected in SD Ura− or YPD nourseothricin (200 mg/liter) media, and the levels of expression of the corresponding Cas9 and dCas9 proteins were detected by Western blotting using Cas9 polyclonal (Clontech) and anti-Flag clone M2 (Sigma) antibodies. *GAL4* and *NRG1* transcriptional modulators were amplified by PCR with primer pairs Up-GAL4/Rev-GAL4 and Up-NRG1/Rev-NRG1, subcloned into intermediary vector pGEM-T, digested with XhoI*/*NotI, and finally inserted into pNRU-dCas9 or pNIM1RX-dCas9 vectors that had been previously digested with XhoI*/*NotI, generating corresponding vectors pNRU-dCas9-Gal4, pNIM1RX-dCas9-Gal4, pNRU-dCas9-Nrg1, and pNIM1RX-dCas9-Nrg1.

10.1128/mSphere.00001-19.3 TABLE S2 List of plasmids used in the work. The main characteristics of the plasmid are indicated, such as the name of the plasmid, the promoter and gene regulated, the parental vector, the marker used, and the integration region in the Candida albicans genome. Download Table S2, XLSX file, 0.01 MB. Copyright © 2019 Román et al. 2019 Román et al. This content is distributed under the terms of the Creative Commons Attribution 4.0 International license.

10.1128/mSphere.00001-19.4 TABLE S3 List of oligonucleotides used in the work. In addition to the sequences, their uses are also indicated in the notes. Colors in the sequence indicate the base changes that introduce a restriction recognition site or a mutation. Download Table S3, XLSX file, 0.01 MB. Copyright © 2019 Román et al. 2019 Román et al. This content is distributed under the terms of the Creative Commons Attribution 4.0 International license.

The gene reporter vector containing a *CAT1* promoter fused to GFP strain pCAT1-GFP was obtained as follows. pDU1-GFP_myc was constructed as follows. *ACT1* promoter was amplified by PCR with Up-pACT1 and Rev-pACT1, digested with SalI/NheI, and cloned into pDU0-L, which had been digested previously with the same pair of enzymes, to generate pDU1-L (44). The click beetle luciferase (CbLUC) open reading frame (ORF) was removed from pDU1-L by cutting with SalI and BglII and replaced by GFP_Myc obtained from SalI/BglII digestion of plasmid pNIM1_MoGFP_carboxi_ca_myc (45). We amplified 1,600 bp containing the coding region of *HIS1* with primers o-up-HIS1-SpeI and o-rev-HIS1-SacII and cloned the result into pDU1-GFP_myc, previously digested with SpeI and SacII, to generate pDH0-GFP_myc. To generate a cloning site, we replaced the 5´ *ARD1* region of pDH0-GFP_myc with the amplicon generated by PCR using primers Up-5 *ARD1*-KpnI and Low-5 *ARD1*-BamHI BswiI and SalI (Table S3) containing the same 5´ *ARD1* region (455 bp) by digestion with KpnI and SalI, generating vector pDH0MGFP_myc. A 1,000-bp fragment containing the putative *CAT1* regulatory region was amplified by PCR with Up-1kbp Pr CAT1 BamHI and Low-1kbp Pr CAT1 BsiWI (Table S3), digested with the indicated enzymes, inserted into plasmid pDH0MGFP_myc, previously digested with BamHI and BsiWI, and treated with alkaline phosphatase (New England Biolabs [NEB]) to generate the final vector pDH8M-GFP_myc. KpnI and SalI digestion was used to force homologous recombination in the *ARD1* locus to generate the pCAT1-GFP tester strain. Transformants were selected in SD His− media and confirmed for GFP expression by Western blotting using anti-GFP antibody and by FC.

### Bioinformatic analysis of CAT1 upstream region.

The 1,594-bp upstream *CAT1* ORF (orf19.6229) (see Fig. S1D in the supplemental material) was analyzed by the use of different software programs for determination of potential regulatory regions. In the text that follows, the numbering refers to this specific region; therefore, “1595” represents the “A” in the ATG starting codon. Neural Network Promoter Prediction (http://www.fruitfly.org/seq_tools/promoter.html) predicted the following stretches of regulatory DNA sequences: positions 124 to 174 (score of 0.91, potential transcriptional start site [TSS] at position 164), 804 to 854 (score of 0.93, potential TSS at 844), and 1389 to 1439 (score of 0.96, potential TSS at 1429). Potential TATA boxes were analyzed with Comet Software (https://zlab.bu.edu/~mfrith/comet/form.html) with a threshold E value of lower than 8, which gave the following results: TATA1 (positions 1398 to 1412, positive strand, score of 0.939, E value of 4.08), TATA2 (positions 131 to 145, positive strand, score of 0.376, E value of 7.53), TATA3 (positions 1111 to 1125, negative strand, score of 0.332, E value of 7.9), and TATA4 (positions 1294 to 1308, negative strand, score of 0.272, E value of 8.44). Combining the two analyses and considering the associated probabilities, the most probable promoter regulatory region would be between positions 1389 and 1398 or between positions 1412 and 1439. Guides were selected in this 1,594-bp region using CHOCHOP software (http://chopchop.cbu.uib.no/index.php).

### sgRNA design, generation and cloning.

We use different approaches for sgRNA design and cloning depending on the strategy used for the sgRNA-expressing method. In one approach, sgRNAs were inserted into a BsmBI cloning site in the corresponding vector, pV1090 or pV1093 (kindly supplied by Vyas et al. [13]). sgRNAs were generated by phosphorylation and annealing of complementary single-stranded DNA (ssDNA) oligonucleotides (see Table S3) and then inserted into the corresponding vector, previously digested with BsmBI, and treated with alkaline phosphatase, calf intestinal (CIP) (NEB). In some cases, we used an NEB Builder HiFi master assembly commercial kit, which allows direct cloning (in one step and with high efficiency) of the sgRNAs whose sequences harbor the desired target sequence (20 nt in length) flanked with a 25-nt sequence that hybridized with the cloning vector. Insertion of the correct sgRNAs was confirmed by sequencing using o-seq-pV1093, 200 bp downstream sgRNA cloning site (Table S3). The corresponding sgRNAs were integrated into the *RP10* locus after KpnI and SacII digestion, and transformants were selected in YPD plus nourseothricin (clonNAT; Werner BioAgents) (200 mg/liter). The correct integration was confirmed by PCR analysis of genomic DNA with 0-to-100 bp of guide cloning site pV1090 up (in pSNR52) and Rev-ORFX-con pv1090 (outside the *RP10* integration site). In the second strategy, as previously described (19), ssDNA sequences incorporated a SapI recognition sequence. After the two-step protocol was performed as previously described, sgRNAs were directly cloned into the pND494 vector digested with SapI and treated with alkaline phosphatase (CIP; NEB). The correct insertion was confirmed by analysis of the loss of the ClaI site in the cloning site. The corresponding sgRNAs were integrated into the *RPS1* locus after StuI digestion, and transformants were selected in SD Ura−.

### Yeast scaffold RNA (scRNA) sequence design.

scRNA sequences with RNA recruitment hairpins were synthetized for C. albicans codon optimization on the basis of the codon usage of four highly expressed C. albicans genes (*HWP1*, *ENO1*, *MRPS9*, and *ACT1*) (GenScript, USA) following the sgRNA sequence described previously (23, 32). The XmaI-AvaI fragment with RNA binding protein MCP fused to the transcriptional activator VP64 sequences was removed from the pUC57-MCP-VP64 MS2 plasmid and accommodated in the pV1093 vector previously digested with XmaI*/*AvaI, generating the pV1093-MCP-VP64 plasmid. Then, the XhoI-SacII fragment with the sgRNA cloning site and recruitment hairpin MS2 sequences from the pUC57-MCP-VP64 MS2 plasmid were exscinded and inserted into pV1093-MCP-VP64 digested with XhoI-SacII to obtain the final vector, pV1093-MS2-MCP-VP64. Finally, the desired sgRNAs were inserted into the BsmBI sgRNA cloning site as previously described. The final plasmids were then digested with KpnI and SacI to force recombination in the *ENO1* locus. Transformants were selected in YPD plus nourseothricin (clonNAT; Werner BioAgents) (200 mg/liter), and correct integration was confirmed by PCR with primers Up-int-ENO1 and Rev-int-pV1093-MCP-VP64-MS2 (Table S3).

### Protein extracts and immunoblot analysis.

All procedures involving cell lysis, protein extraction, gel electrophoresis, and transfer to nitrocellulose membranes were performed as previously described (46, 47). Protein extracts were measured at *A*_280_ to equalize the amounts of protein loaded for Western blot analysis, and the blots were probed with anti-GFP, anti-Flag clone M2 (Sigma), or anti-Cas9 (Clontech). Western blots were developed according to the instructions of the manufacturer (Amersham Pharmacia Biotech) using a Hybond ECL kit.

### Flow cytometry analysis.

Epifluorescence microscopy images were obtained by the use of an Eclipse TE2000-U inverted microscope (Nikon) coupled with an Orca C4742-95-12 ER charge-coupled-device camera (Hamamatsu). Image capture and processing were performed with AquaCosmos Imaging System 1.3 software. A Guava EasyCyte cytometer and InCyte software (Millipore) were used for flow cytometry and qualitative and quantitative analysis of GFP fluorescence. Data processing and analysis were done by using FlowJo software.

### Statistical analysis.

Statistical differences between two groups were calculated using Student’s two-tailed unpaired *t* tests, correcting for multiple comparisons using the Holm-Sidak method, with α= 0.05. Computations were performed with the assumption that all rows were sampled from populations with the same standard deviation.
