# Supplementary figures and images for: Implementation of a CRISPR-Based System for Gene Regulation in Candida albicans
Source: mSphere. 2019 Feb 13;4(1):e00001-19. doi: 10.1128/mSphere.00001-19 (PMC6374588; doi:10.1128/mSphere.00001-19)

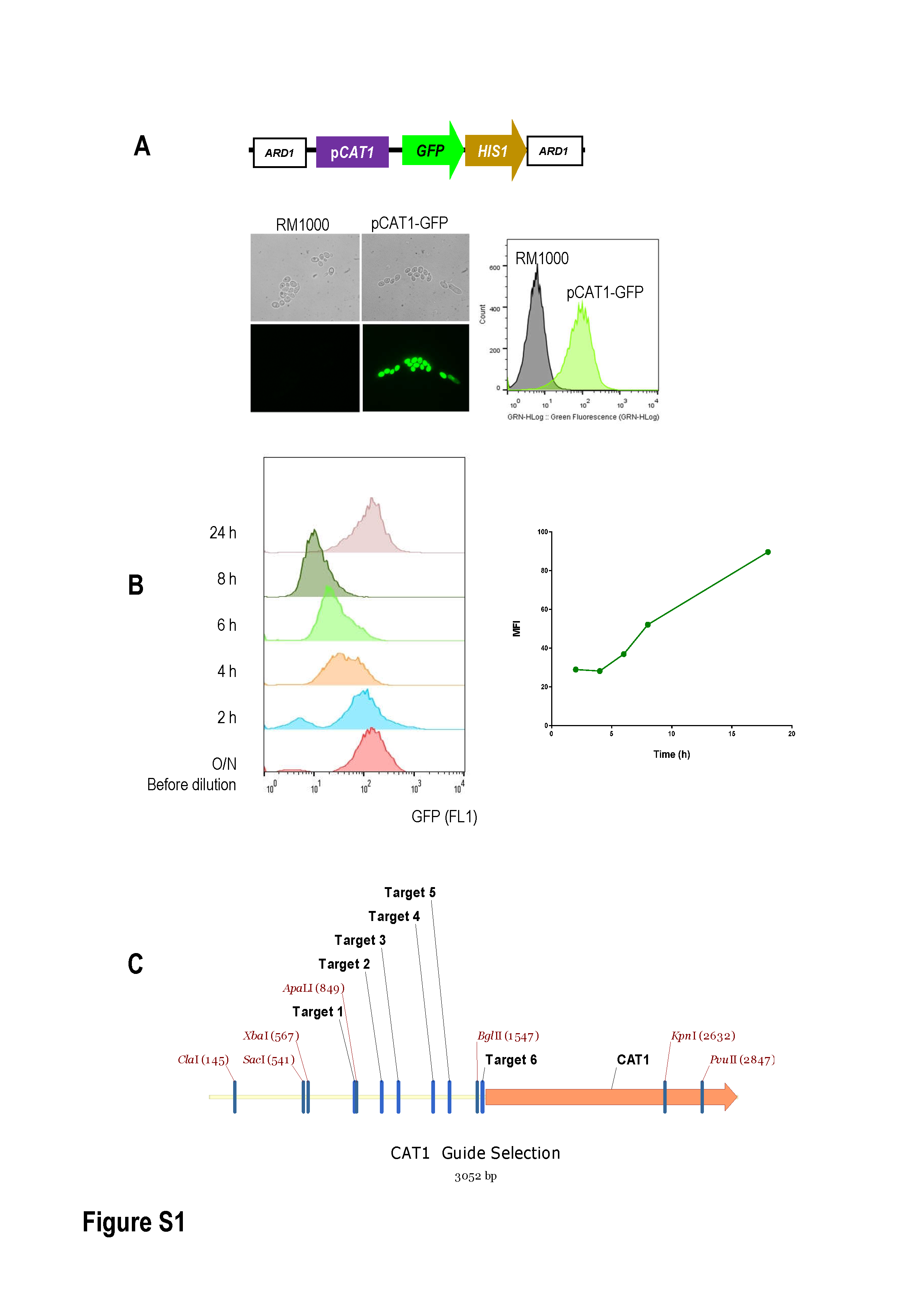

Supplement: FIG S1 [file mSphere.00001-19-sf001.tif]
